# Spatial contribution of hippocampal BOLD activation in high-resolution fMRI

**DOI:** 10.1038/s41598-019-39614-3

**Published:** 2019-02-28

**Authors:** Yoshifumi Abe, Tomokazu Tsurugizawa, Denis Le Bihan, Luisa Ciobanu

**Affiliations:** Commissariat à l′énergie atomique et aux energies alternatives, DRF, Joliot, NeuroSpin, Paris-Saclay University, Gif-sur-Yvette, France

## Abstract

While the vascular origin of the BOLD-fMRI signal is established, the exact neurovascular coupling events contributing to this signal are still incompletely understood. Furthermore, the hippocampal spatial properties of the BOLD activation are not elucidated, although electrophysiology approaches have already revealed the precise spatial patterns of neural activity. High magnetic field fMRI offers improved contrast and allows for a better correlation with the underlying neuronal activity because of the increased contribution to the BOLD signal of small blood vessels. Here, we take advantage of these two benefits to investigate the spatial characteristics of the hippocampal activation in a rat model before and after changing the hippocampal plasticity by long-term potentiation (LTP). We found that the hippocampal BOLD signals evoked by electrical stimulation at the perforant pathway increased more at the radiatum layer of the hippocampal CA1 region than at the pyramidal cell layer. The return to the baseline of the hippocampal BOLD activation was prolonged after LTP induction compared with that before most likely due vascular or neurovascular coupling changes. Based on these results, we conclude that high resolution BOLD-fMRI allows the segregation of hippocampal subfields probably based on their underlying vascular or neurovascular coupling features.

## Introduction

Functional magnetic resonance imaging (fMRI) based on blood oxygen-level dependent (BOLD) signal^1^ is a powerful tool to non-invasively visualize brain activation^2^. The BOLD-fMRI method relies on measuring changes in blood flow and blood oxygenation level, representing therefore an indirect indication for modified neuronal activity^3,4^. Moreover, the BOLD signal is not cell type specific as it reflects neural as well as glial activation through the underlying neuron-glia-vascular interactions^5–7^. Today, the exact neurovascular coupling events contributing to the BOLD signal are not known. Furthermore, the hippocampal spatial properties of the BOLD activation are not elucidated, although electrophysiological techniques and other neuroimaging approaches with calcium indicators and voltage-sensitive dyes have already revealed the precise spatial patterns of neural activity^8–11^.

The hippocampus has a unique and layered structure and it is anatomically subdivided into six areas: CA1, CA2 and CA3, the dentate gyrus (DG), the subiculum (Sub) and the entorhinal cortex (EC). The inputs to the EC come from the associate cortex and sequentially pass through the DG, CA3, CA1 and Sub and back to the EC forming the main hippocampal system. This hippocampal system consists of two specific pathways: tri-synaptic (EC → DG → CA3 → CA1) and mono-synaptic (EC → CA1). The CA1 region has typical layers: the oriens layer with abundant axons of CA1 pyramidal cells, the pyramidal layer having abundant neuronal cell bodies, the radiatum layer with abundant neuronal dendrites projected mainly from CA3 pyramidal cells via the Schaffer Collateral pathway, and the lacunosum molecular layer with abundant neuronal dendrites projected from CA3 via the Schaffer Collateral pathway and from EC via the perforant pathway. The radiatum layer and the lacunosum molecular layer of the CA1, as well as the molecular layer of DG, are rich in blood vessels and capillaries^12,13^ and allow the analysis of the fMRI spatial patterns in high-resolution studies.

The in-plane resolution of previous hippocampal fMRI studies in rodents range from 200 to 250 μm^14–20^. Furthermore, during preprocessing voxels are typically spatially smoothed with a full width at half maximum (FWHM) Gaussian kernel of three times the voxel size, further decreasing the effective spatial resolution. Hence, these past hippocampal fMRI studies were not able to visualize the exact location of the BOLD activation within the different sublayers. Small animal fMRI at ultra-high magnetic fields is possible^21^ and has the potential to increase the spatial resolution and to resolve smaller brain structures.

In this study, we performed ultra-high field fMRI acquisitions to address the spatial contribution of the hippocampal BOLD activation in a rat model. The hippocampal BOLD activation evoked by electrical stimulation at the perforant pathway was measured in the CA1 region including the radiatum, the lacunosum molecular layer and the pyramidal cell layer. In addition, we evaluated the spatial characteristics of the hippocampal BOLD activation when a long-lasting increase in synaptic efficacy was induced by long-term potentiation (LTP). The group of Angenstein *et al*. has previously investigated the relationship between the hippocampal BOLD response patterns and the electrical stimulation patterns at the perforant pathway^14–17^. Two other groups have reported the expansion of the hippocampal BOLD responses to other regions after LTP^18,19^. In this study, we have focused on the spatial pattern of the BOLD response at the hippocampal CA1 region and the spatial properties of the BOLD response within the hippocampus before and after LTP induction.

## Results

### Electrophysiological confirmation of LTP induction

**Figure 1:**
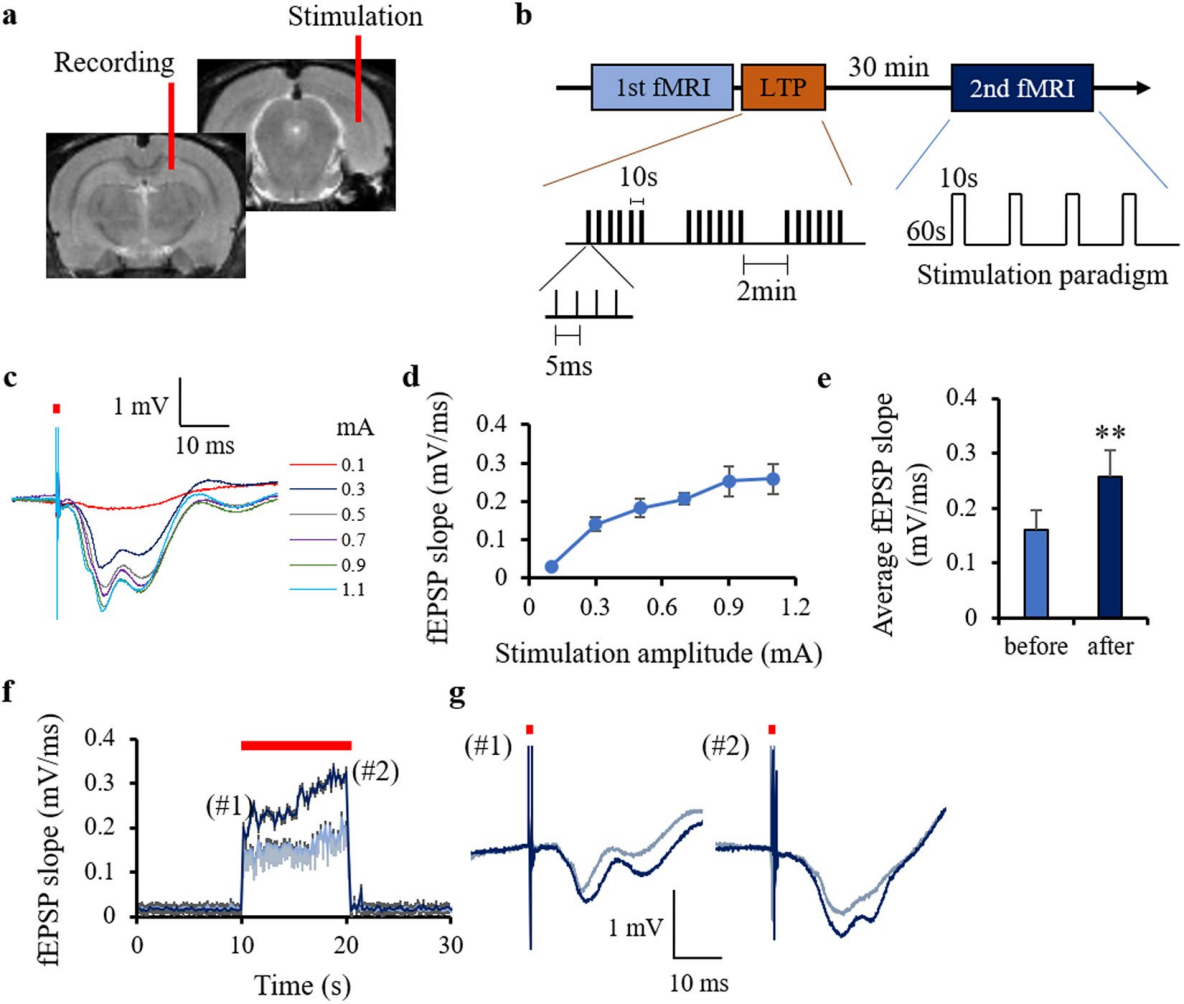
Experimental design and electrophysiology data. (**a**) Schematic showing the positions of the electrical stimulation (perforant pathway) and the LFP recording (radiatum layer of CA1) electrodes. The recording electrode was removed before the fMRI study. (**b**) Diagram showing the timing of the BOLD data acquisition: before and 30 minutes after the LTP induction. (**c**) Representative LFP responses at the radiatum layer (stimulation intensities 0.1–1.1 mA). The red bar shows one pulse of electrical stimulation. (**d**) fEPSP slope at each stimulation amplitude. (**e**) Average fEPSP slope before and after LTP. (**f**) Stimulation induced changes in fEPSP slope before and after LTP induction. The red bar in (**f**) shows periods of electrical stimulation (5 Hz). (**g**) Representative LFP responses at the first stimulation pulse (#1) and the last pulse (#2) before and after LTP induction. ^**^p < 0.01 (Student’s t-test). The plots exhibit mean ± sem.

At first, we confirmed that LTP occurred in the CA1 region after high-frequency stimulation of the perforant pathway as the slope of the field excitatory postsynaptic potential (fEPSP) evoked by 10 s, 5-Hz stimulation increased 30 min after LTP induction (Fig. 1e–g). No afterdischarge response was detected for these stimulation parameters as, at the end of the stimulation, the fEPSP immediately returned to the baseline.

### Spatial contribution of hippocampal BOLD activation evoked by stimulation of the performant pathway

**Figure 2:**
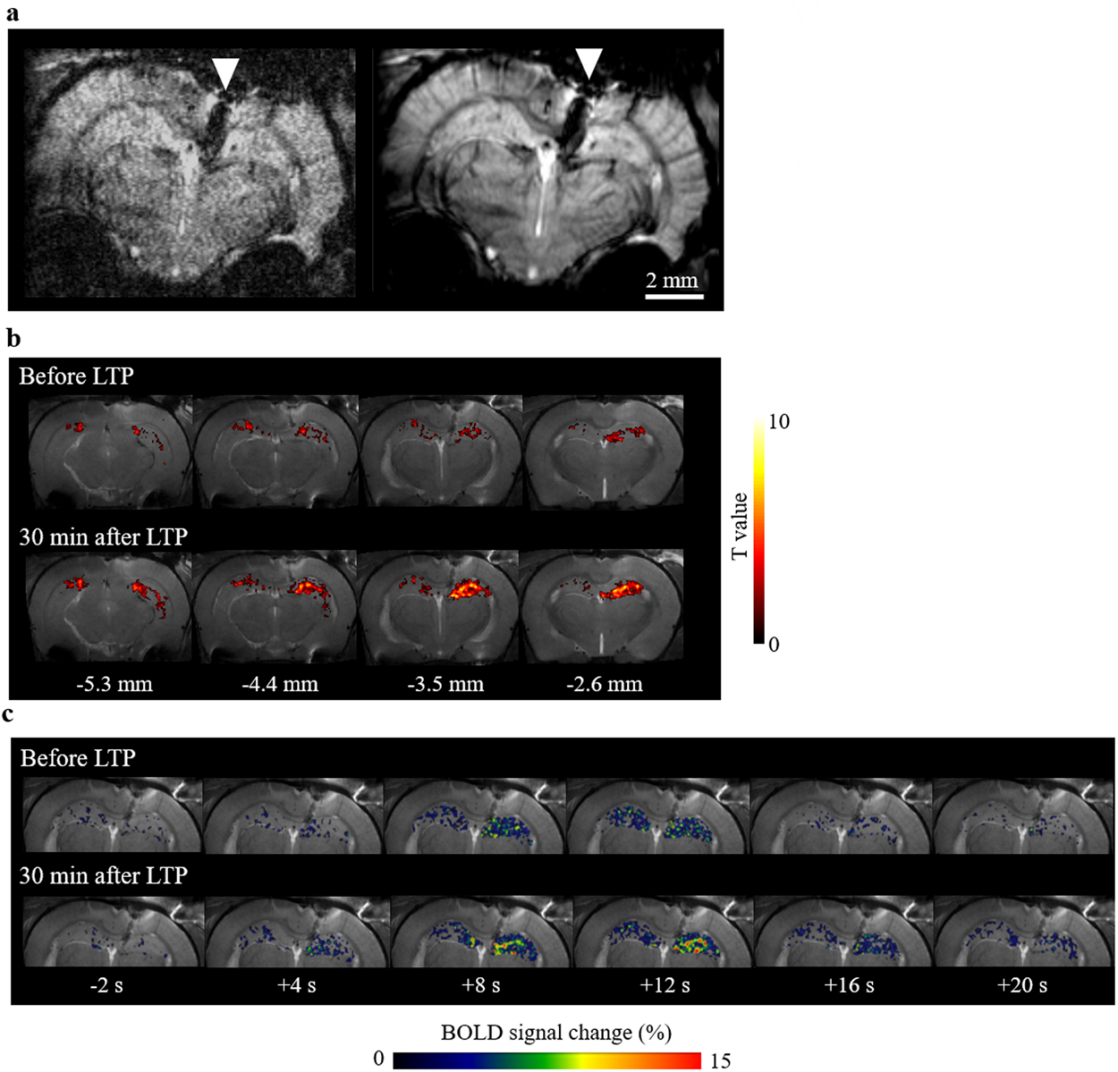
Hippocampal BOLD activation maps before and after LTP induction. (**a**) Representative EPI images: single scan (left) and average image (right). The white triangle points to an artifact induced by the presence of the recording electrode. (**b**) BOLD activation maps produced using SPM before and after LTP induction. The hot color map signifies BOLD signal increase. The digits shown below the maps represent the distance of each slice from the bregma (**c**) Temporal BOLD changes, averaged over four stimulation trains, at −3.5 mm from the bregma before and after LTP induction. The electrical stimulation at the performant pathway was performed for 10 s. In this figure, all data are from the same animal.

**Figure 3:**
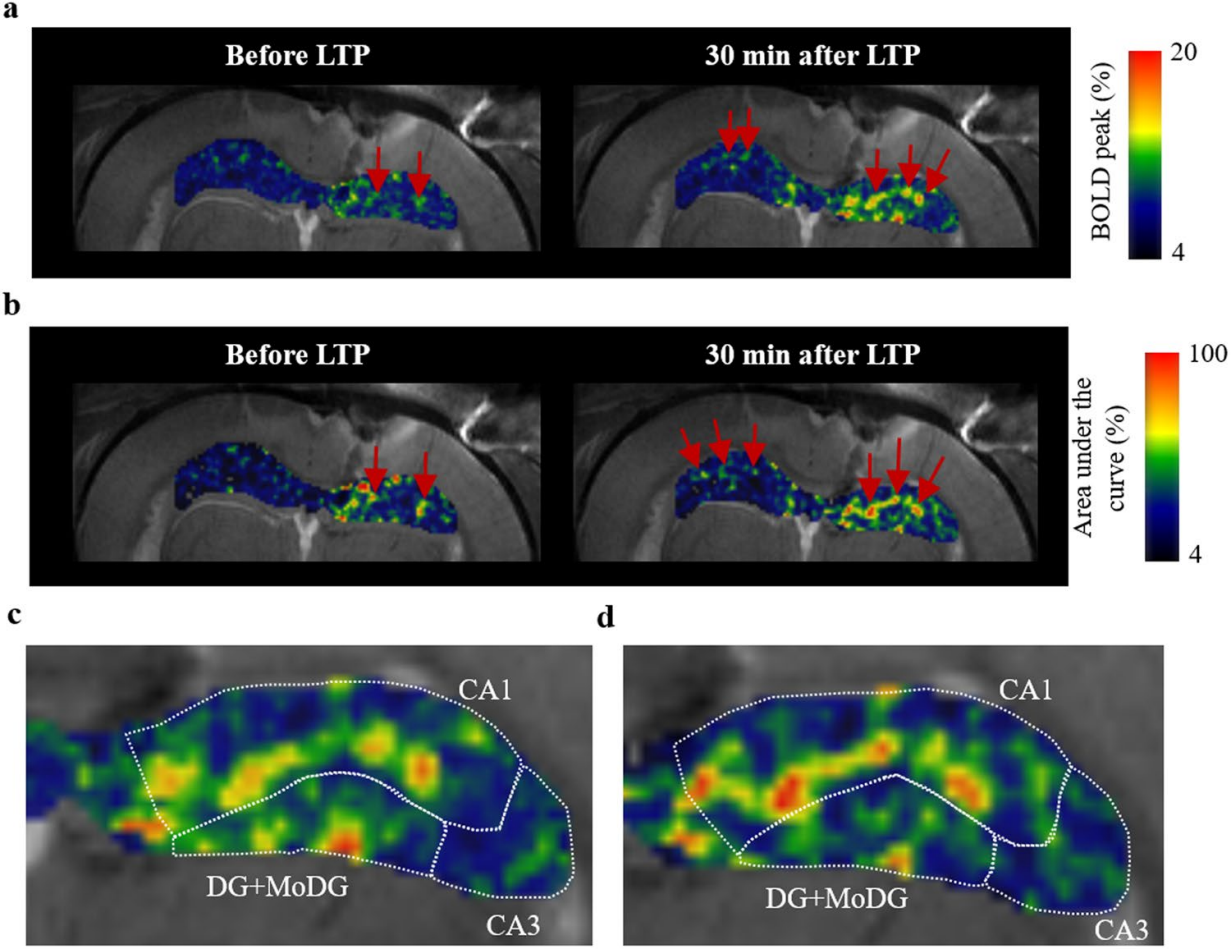
Maps of BOLD peak and BOLD area under the curve. Maps of BOLD peak intensity (**a**) and area under the curve (**b**) before and after LTP induction. The peak intensity and the area under the curve are averages over four stimulation periods. The red arrows point to regions manifesting a significant increase in the BOLD peak intensity or area under the curve at the radiatum layer of CA1. Zoomed images of the BOLD peak map (**c**) and the area under the curve map (**d**) within the ipsilateral hippocampus after LTP induction.

Representative BOLD images (raw GE-EPI images and the average image) are shown in Fig. 2a. Hippocampal BOLD activation evoked by10 s, 5-Hz stimulation at the perforant pathway was obtained bilaterally and this activation increased 30 min after LTP induction (Fig. 2b). Time-series of the BOLD activation showed that the BOLD signal increased after LTP induction, compared to that before LTP induction (Fig. 2c). The BOLD peak intensity and the area under the curve, reflecting the total activity^22,23^, were higher at the radiatum layer of CA1 (Fig. 3). After LTP induction, the response clearly increased at the radiatum layer of CA1 with regions showing higher BOLD peak intensity and area under the curve expanding within the hippocampus (Fig. 3). This result indicates that higher BOLD activation evoked by stimulation at the perforant pathway occurred particularly in a specific region, the radiatum layer of CA1.

### Characterization of BOLD time courses in several hippocampal regions

**Figure 4:**
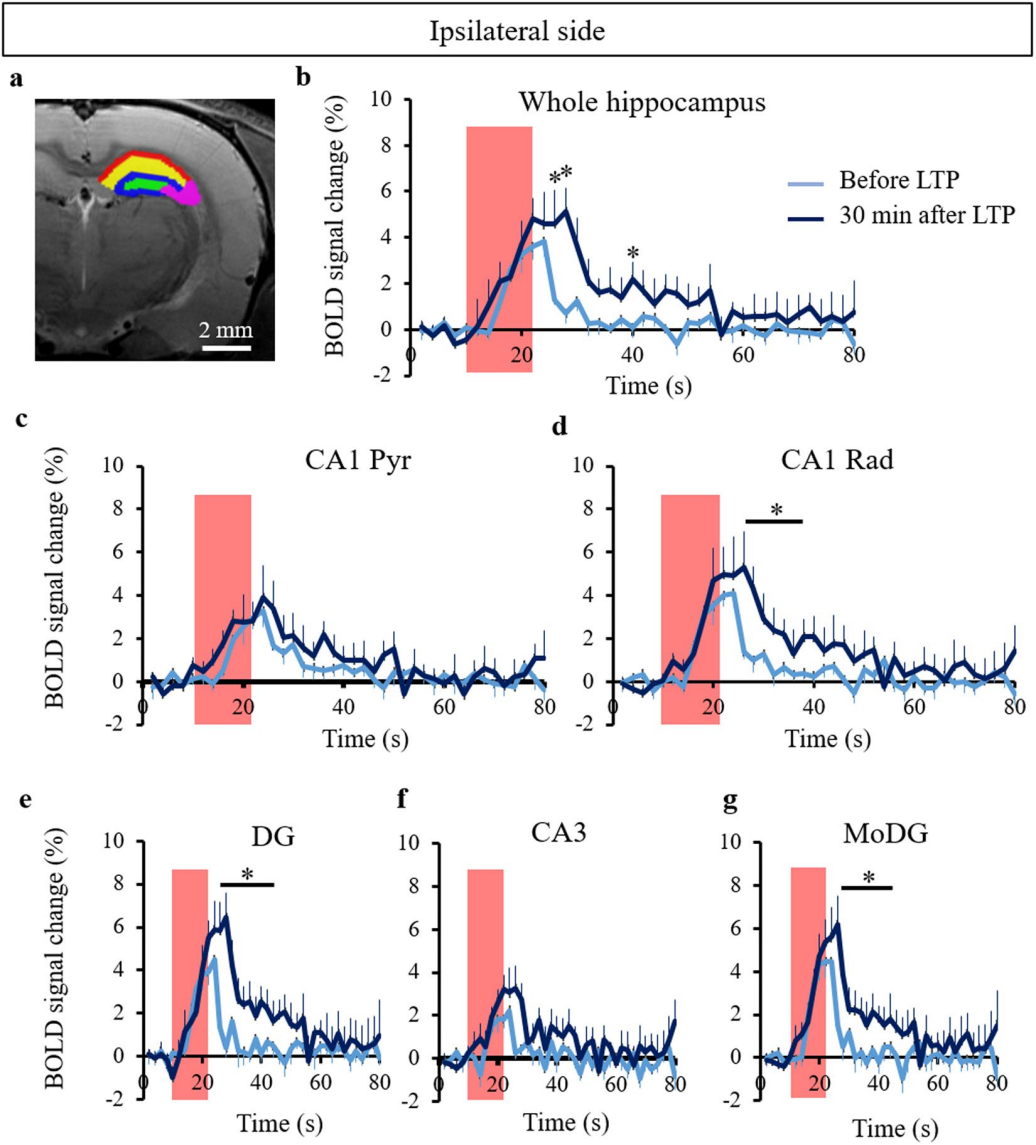
Time course of the BOLD response in the ipsilateral hippocampus. (**a**) Color coded ROIs (green: dentate gyrus (DG), purple: CA3, blue: molecular layer of DG (MoDG), yellow: radiatum layer of CA1 (CA1 Rad), and red: pyramidal layer of CA1 overlaid on the T2 weighted structural image. BOLD time courses in the entire ipsilateral hippocampus (**b**), CA1 Pyr (**c**), CA1 Rad (**d**), DG (**e**), CA3 (**f**), and MoDG (**g**) before and after LTP induction. The red boxes correspond to periods of electrical stimulation at the perforant pathway. The bar plots exhibit mean ± sem. ^*^p < 0.05 (paired t-test). The interaction between BOLD time course and LTP effects (two-way repeated ANOVA) showed no significance.

**Figure 5:**
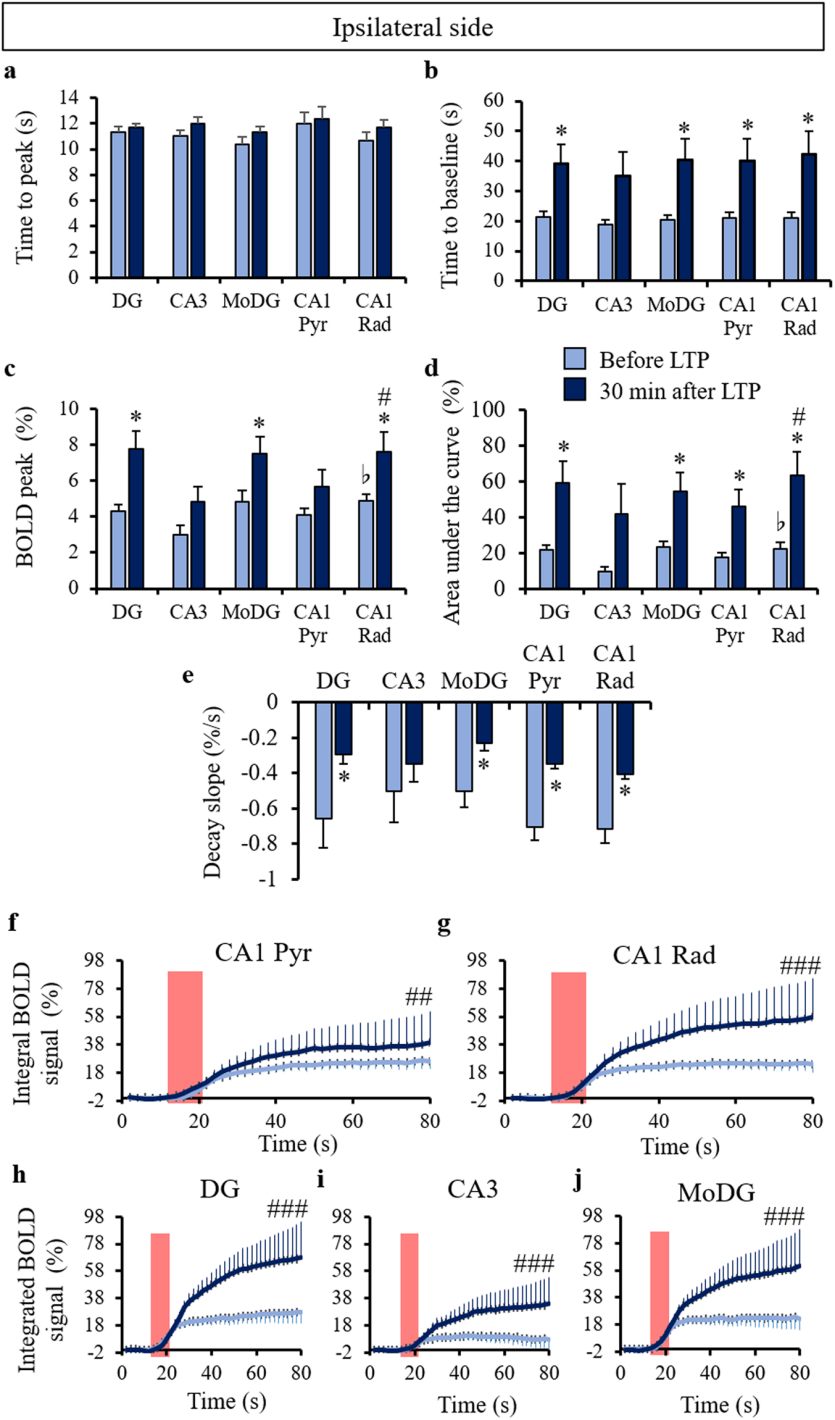
BOLD response parameters in the ipsilateral hippocampus. The time to peak (**a**), time to baseline (**b**), peak intensity (**c**), area under the curve (**d**), and decay slope were measured using the same ROIs as in Fig. 4a. ^*^p < 0.05 (paired t-test after versus before LTP at each region), ^b^p < 0.05 (paired t-test Rad versus Pyr before LTP), and ^#^p < 0.05 (paired t-test Rad versus Pyr 30 min after LTP). The integrated BOLD responses shown at CA1 Pyr (**f**), CA1 Rad (**g**), DG (**h**), CA3 (**i**), and MoDG (**j**) before and after LTP induction. The red boxes represent electrical stimulation periods at the perforant pathway. ^##^p < 0.01, ^###^p < 0.001 (LTP effect of the two-way repeated ANOVA). The bar plots exhibit mean ± sem.

Based on the results presented previously showing that the highest BOLD activation occurred at the radiatum layer of CA1, we identified DG, CA3, the molecular layer of DG (MoDG), the pyramidal layer and the radiatum layer of CA1 as regions in which to investigate the characteristics of the hippocampal BOLD activation in detail (Fig. 4a). We confirmed that the BOLD signal increased in the entire ipsilateral hippocampal region after LTP induction (Fig. 4b). We obtained the entire BOLD time courses with 4 stimulus epochs before and after LTP induction (Supplementary Fig. S2). After LTP induction, the BOLD responses in the decay period at the ipsilateral and contralateral sides were significantly higher at the radiatum layer than those before LTP induction (Fig. 4d and Supplementary Fig. S3), while they did not show a significant increase at the pryramidal layer of the ipsilateral and contralateral sides (Fig. 4c and Supplementary Fig. S3). The BOLD peak intensity also increased significantly at the radiatum layer of the ipsilateral side after LTP induction but not at the pyramidal layer (Fig. 5c). The time to baseline, the area under the curve, and the decay slope showed increases in both the radiatum and pyramidal layers of the ipsilateral side (Fig. 5b,d,e). There was no difference in time to peak between the regions investigated (Fig. 5a). The integrated BOLD area significantly increased in all regions of the ipsilateral side after LTP induction (Fig. 5f–j). These results indicate that LTP-induced BOLD increase occurred more at the radiatum layer of the CA1 than at the pyramidal layer and that the return the base line was delayed. In addition, the BOLD peak intensity and the area under the curve at the radiatum layer of the ipsilateral side were significantly higher than those at the pyramidal layer (Fig. 5c,d). Similar results were obtained when comparing two processing approaches, with and without smoothing, as demonstrated in Supplementary Fig. S4.

Similar to the CA1 region, the BOLD responses in the decay period after LTP induction were significantly higher than those before LTP induction in the DG and MoDG but not CA3 of the ipsilateral side (Fig. 4e–g). The BOLD peak, time to baseline, the area under the curve, and the decay slope increased at DG and MoDG but not CA3 of the ipsilateral side after LTP induction (Fig. 5b–e). There was no difference in the time to peak in these regions (Fig. 5a). In the contralateral hippocampus, the BOLD time course significantly increased only at the radiatum layer of CA1 after LTP induction (see Supplementary Fig. S3). Although the time to peak, the time to baseline, the BOLD peak intensity, the area under the curve, and the decay slope did not significantly change in the contralateral hippocampus after LTP induction, the time to baseline and the area under the curve displayed a tendency to increase after LTP induction in the contralateral hippocampus (see Supplementary Fig. S3).

## Discussion

In this study, we used high-resolution hippocampal fMRI evoked by electrical stimulation at the perforant pathway to investigate the spatial contributions of different hippocampal regions to the BOLD signal. In agreement with previous literature reports, we observed an increase of neural activity in the hippocampus after the electrical stimulation at the performant pathway^8–11^. An increased evoked potential was measured at CA1, most likely propagated from DG via the trisynaptic pathway DG - CA3 - CA1 (Supplementary Fig. S1a). We also found that the hippocampal BOLD signals increased more at the radiatum layer of the hippocampal CA1 region than at the pyramidal layer. Given that the BOLD signal reflects changes in blood volume and oxygenation, both in veins and arteries^24,25^, one can assume that the differences found between the two CA1 regions reflect the difference in their vascularization, with the radiatum layer exhibiting a higher density of blood vessels and capillaries^12,13,26^. Another possible explanation could be the difference in astrocytic density at the two CA1 layers^27^ as astrocytic activity also modulates the BOLD signal response^7^. From our BOLD experiments, it is not possible however to identify which cells (vascular cells, neurons or astrocytes) are most important for generating the BOLD response patterns observed. Interestingly, electrical activity imaging of hippocampal slices using voltage-sensitive dyes also showed spatial patterns with increased neural activity at the radiatum layer compared to that at the pyramidal layer^10,11^. The current-source density analysis with multi-unit electrical recordings revealed that neural signals were inputted at the radiatum layer from Schaffer Collateral as well as the perforant path^8,9^. Intrinsic optical signal imaging in the hippocampal slices exhibited similar spatial patterns^28^. All these data on the regional CA1 activation are similar with our current results of the hippocampal BOLD activation pattern, suggesting that the different subregions identified by fMRI, which reflect vascular and/or neurovascular coupling features, may colocalize with precise activation regions. Simultaneous BOLD and multi-unit electrical recordings are necessary to further confirm this.

It is well known that LTP induces an increase in neural activation. High-frequency stimulation at the perforant pathway can be used to induce LTP at the DG, the CA3, and the CA1 regions^29–31^. Our results revealed that the BOLD signal increased both in the ipsilateral and contralateral DG and CA1 regions after LTP induction, although this increase was not significant in the latter. Other groups demonstrated that LTP induction expanded the BOLD activation area to the prefrontal and perirhinal cortex, the nucleus accumbens, and the anterior olfactory nucleus^18,19^, regions which were not investigated in the current study.

Our results showed increases in the return to baseline time, the area under the curve, and the integrated BOLD signal in the ipsilateral DG, MoDG, CA1 regions after LTP induction and no change in the time to peak of the BOLD signal, indicating that the BOLD activation was delayed returning to the baseline. While in some conditions afterdischarge can lead to prolonged BOLD signals^20^, no afterdischarge was not observed for the experimental conditions used here. This suggests that the cause of the prolonged BOLD response is not neural plasticity induced by LTP, but instead an increased vascular and/or astrocytic activity^7,32^. A decoupling between neuronal and hemodynamic signals is possible as pointed by O’Herron *et al*.^4^. Moreover, the temporal profile of the vascular response (increased CBV) evoked by neural activity is different from that of neural activity itself^33^, typically with a longer return to baseline^34^. Another explanation for the prolonged BOLD signal could be a change in astrocytic activity. Astrocytes cooperate with neurons and regulate the neuronal activity and the brain blood flow^5,35^, which can influence the BOLD signal prolongation. In addition, astrocytic activity is LTP modulated and astrocytes themselves showed an LTP like response^36,37^. A previous fMRI study combined with calcium imaging demonstrated that the BOLD activation coupled with astrocytic activity, which was likely related to metabotropic glutamate receptor signaling^5^, was prolonged more than that uncoupled with astrocytic activity^7^. These results suggest that a change in astrocytic activity coupled with the vascular response can cause the delayed return to the baseline of the BOLD activation observed after LTP, but their exact function in neurovascular coupling remains unclear. Different factors associated with vascular and astrocytic activity such as energy metabolism, neurotransmitter trafficking and recycling, ion homeostasis^5,35^ can also influence the BOLD signal change observed. Further investigations are needed to precisely identify the cause of the change of the BOLD response after LTP induction.

## Conclusion

High resolution, ultra-high field BOLD functional MRI allows the segregation of hippocampal subfields based of their differences in the underlying vascular and/or neurovascular coupling features. It remains to be shown whether these features co-localize with differential neuronal activities using alternative, invasive approaches.

## Methods

### Animals

12 male Wister rats (180–200 g, Janvier Labs, Saint Berthevin, France) were used in this study; 6 rats were used for the fMRI study and 6 rats for LFP recordings of LTP induction. The rats were housed two per cage under controlled light (7:00–19:00) conditions and were given free access to water and food. All animal procedures used in the present study were approved by the *Comité d’Ethique en Expérimentation Animale, Commissariat à l’Energie Atomique et aux Énergies Alternatives, Direction des Sciences du Vivant (Fontenay-aux-Roses, France)* and by the *Ministère de l’Education Nationale de l’Enseignement Supérieur de la Recherche (France)* under reference *APAFIS#2393–2015102211447539v2 and were conducted in strict accordance with the recommendations and guidelines of the European Union (Directive 2010/63/EU) and the French National Committee (Décret 2013-118)*.

### Surgery and LFP recording

The LFP recordings were performed before the fMRI study outside the MRI scanner. The animals were initially anesthetized with 1.5–2.0% isoflurane and two tungsten micro electrodes were inserted under stereotaxic conditions. A monopolar electrode (1.0 MΩ, 3-μm tip and 125- μm shaft diameter; MicroProbe, MD, USA) was inserted into the CA1 radiatum layer (−2.3 mm ML, −4.1 mm AP, −2.3 mm DV from the bregma) for LFP recordings and a home-made bipolar electrode (5μm diameter with PFA-coat; A-M Systems, WA, USA) was inserted into the post subiculum including the perforant pathway (−4.1 mm ML, −7.5 mm AP, −3.0 mm DV) for stimulation (Fig. 1a). After the surgery, the anesthesia was changed from isoflurane to 0.05 mg/kg/h s.c. medetomidine (Domitor; Pfizer Animal Health, New York, NY, USA). A catheter was inserted subcutaneously allowing a continuous infusion of medetomidine via a syringe pump (Harvard Apparatus; Holliston, MA, USA). The medetomidine infusion was started after a subcutaneous bolus of 0.05 mg/kg. During the surgery and the LFP recordings, the animal’s temperature was maintained at 37 °C using a heating pad (DC temperature controller; FHC Inc., Bowdoin, ME, USA). The recording electrode was connected to a differential AC amplifier (AM systems, Sequim, WA, USA) via a Model 1700 head stage (AM systems, Sequim, WA, USA). The reference electrode (connected to ground) was inserted into the scalp. The LFP data was recorded using a Powerlab 8/35 with LabChart (AD Instruments; Dunedin, New Zealand) with a bandpass of 0.1–1000 Hz and digitized at 20 kHz. The 50 Hz noise from the power line was eliminated from the signal using selective filters. The fEPSP responses at hippocampal CA1 region were evoked with square pulses (0.3 ms pulse width, 0.1–1.1 mA intensity) at the perfornat pathway (Fig. 1c). Standard input-output curves were generated by plotting the slope of fEPSP (Fig. 1d). The stimulation intensity for the fMRI study and the LTP induction was the one that produced 50% of maximal amplitude of the fEPSP. The average intensity (mean ± sem) over the animals used for the fMRI study was 0.41 ± 0.03 mA. After recording the fEPSP, the electrode for recording at CA1 was removed and the stimulation electrode at the perforant pathway was fixed with dental cement (GC Unifast Trad; GC CO., Tokyo, Japan) and an adhesive (Super-Bond C and B; Sun Medical CO., LTD., Shiga, Japan).

The continuous evoked fEPSPs induced by 5 Hz stimulation for 10 seconds were recorded before and 30 minutes after LTP induction using different animals from those used in the fMRI study. LTP was induced by high-frequency stimulation at the perforant pathway with episodes of six trains of pulses (each train was delivered at 200 Hz and lasted 40 ms, with four pulses per train and trains delivered every 10 s, repeated three times with pauses of 2 min between episodes^18^ (Fig. 1b). The electrophysiology data was analyzed using an in-house MATLAB program.

### Stimulation protocol for the fMRI study

The fMRI block design used consisted of 4 periods of 10 s stimulation epochs at 5 Hz and current intensities evoking half-maximal fEPSP in the radiatum layer of CA1, followed by a resting epoch of 60 s (Fig. 1b). The fMRI data was obtained before and 30 minutes after LTP induction (Fig. 1b).

### MRI acquisitions

All fMRI acquisitions were performed on a 17.2 T MRI scanner (Bruker BioSpin; Ettlingen, Germany) equipped with 1 T/m gradients. The radio frequency (RF) transceiver was a surface coil consisting of a single loop (inner diameter of 30 mm; Bruker, BioSpin; Ettlingen, Germany). The animals, fixed with ear pins and a tooth bar on an MRI bed, were anesthetized with a continuous infusion of 0.05 mg/kg/h medetomidine. The respiration rate and the rectal temperature were monitored and ensured to stay in normal ranges during scanning. A mixture of air and 20% oxygen was supplied to the animals.

Good B0 homogeneity was ensured through automatic iterative FASTMAP methods (ParaVision 5.1). BOLD images, with focus on the hippocampus (−2.6 to −6.2 mm from the bregma), were acquired using a gradient echo EPI sequence with the following parameters: TR = 1000 ms, TE = 11 ms, N segments = 2, bandwidth = 400 kHz, flip angle = 90°, FOV = 1.8 × 1.25 cm^2^, matrix = 180 × 125, number of slices = 5, slice thickness = 0.8 mm, slice gap = 0.1 mm, spatial resolution = 100 × 100 × 800 μm^3^, temporal resolution = 2 s, dummy scans = 3, number of repetitions = 165, scan time = 5.5 min. T2 weighted structural images were also acquired with the following parameters: 2D-relaxation enhancement (RARE) sequence; TR = 2000 ms, TE_eff_ =  22 ms, RARE factor = 4, FOV = 1.8 × 1.25 cm^2^, matrix = 180 × 125, number of slices = 5, slice thickness = 0.8 mm, slice gap = 0.1 mm, resolution = 100 × 100 × 800 μm^3^, number of averages = 4, scan time = 6 min.

### fMRI data analysis

SPM8 software (Welcome Trust Centre for Neuroimaging, London, UK) and in-house software written in Matlab were used for data preprocessing and statistical analysis. Image preprocessing was performed individually for each animal. Time-series fMRI images were realigned to correct for residual head motion and co-registered to the reference structural RARE image. The fMRI images were smoothed with a full width at half maximum (FWHM) Gaussian kernel of 150 μm (to make sure that the smoothing process did not induce undesirable effects such as shifting of activation we also analyzed our data without smoothing).

General linear modeling in SPM8 was applied to individually generate BOLD activation maps with a significance of p < 0.001 (uncorrected). Because the BOLD activation was obtained only in the hippocampus the rest of the analysis was performed by applying a hippocampal mask, which was manually drawn based on the RARE image. Time-series BOLD activation maps were individually calculated with respect to the baseline averaged over the 10s-period prior to stimulation and were averaged over the 4 stimulation periods. The BOLD peak intensities, defined as the maximum signal change with respect to baseline, were identified and averaged over the 4 stimulation periods. The area under the curve was calculated by summing up the BOLD signal change for each time point from the onset of stimulation to the time when the BOLD signal returned to the baseline, and then averaging over the four stimulation periods. We defined the time to peak as the time to reach the maximum positive BOLD signal and the time to baseline as the time after which the BOLD signal returned to the baseline, both measured from the onset of stimulation. The decay slope was defined as the slope calculated from the time to reach the peak to the time to reach the base line.

Regions of interest (ROIs) corresponding to the dentate gyrus (DG; including the granule cell layer and hilus), the CA3 (including the pyramidal layer and the radiatum layer), MoDG, the pyramidal layer of CA1 (CA1 Pyr; including the pyramidal layer and the oriens layer), and the radiatum layer of CA1 (CA1 Rad; including the radiatum layer and the lacunosum moleculare layer) were drawn on one slice, which did not present artifacts created by the insertion of the electrode, by referring to the Paxinos and Watson rat brain atlas^38^ (Supplementary Fig. S1b). The BOLD signals in these ROIs were individually extracted using MarsBar (MRC Cognition and Brain Sciences Unit, Cambridge, UK). The sizes of these ROIs were 123, 210, 115, 117, and 182 voxels for DG, CA3, MoDG, CA1 Pyr, and CA1 Rad, respectively.

### Statistics

The statistical analysis was performed in Matlab and Excel using Student t-tests, paired t-tests or analysis of variance (ANOVA) followed by post-hoc t-tests. A p value of 0.05 was considered the threshold for significance. The results are reported as mean ± sem.

## Supplementary information

Spatial contribution of hippocampal BOLD activation in high-resolution fMRI
