# Supplementary material for: Spatial contribution of hippocampal BOLD activation in high-resolution fMRI
Source: Sci Rep. 2019 Feb 28;9:3152. doi: 10.1038/s41598-019-39614-3 (PMC6395694; doi:10.1038/s41598-019-39614-3)
Supplement: Supplementary file 1 — Spatial contribution of hippocampal BOLD activation in high-resolution fMRI [file 41598_2019_39614_MOESM1_ESM.doc]

Supplementary information

**Title:** Spatial contribution of hippocampal BOLD activation in high-resolution fMRI

**Authors:** Yoshifumi Abe^1^, Tomokazu Tsurugizawa^1^, Denis Le Bihan^1^, and Luisa Ciobanu^1^

^1^NeuroSpin, Bât 145, Commissariat à l'énergie atomique et aux énergies alternatives, Point Courrier 156, 91191 Gif-sur-Yvette, France.


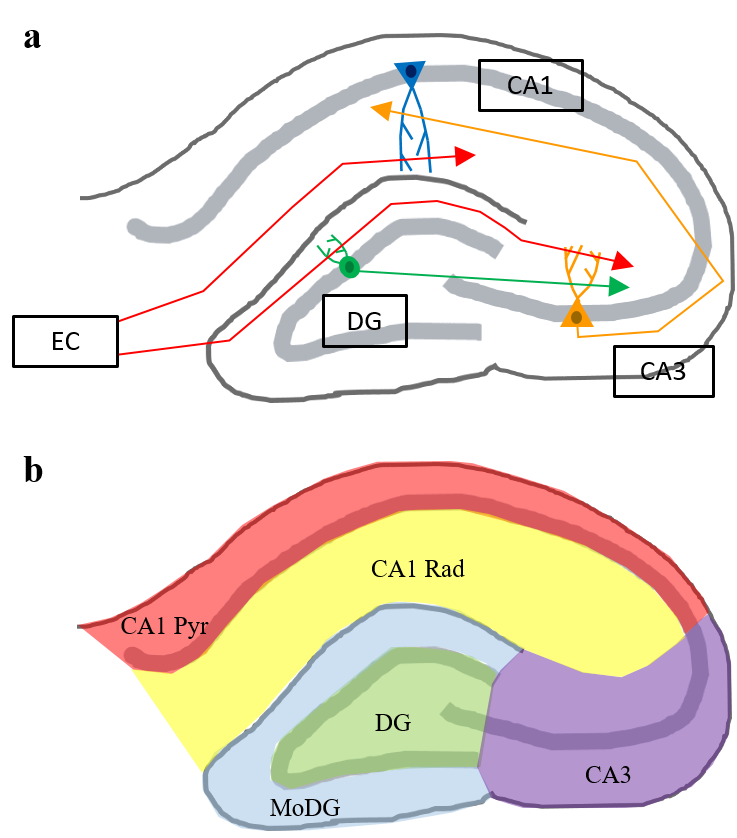


**Supplementary Fig. S1** **Diagram depicting the hippocampal pathways and sub-fields.**

(a) The hippocampus pathway. (b) Color coded regions (green: dentate gyrus (DG), purple: CA3, blue: molecular layer of DG (MoDG), yellow: radiatum layer of CA1 (CA1 Rad), and red: pyramidal layer of CA1 (CA1 Pyr) correspondingto the ROIs in Fig. 4a.


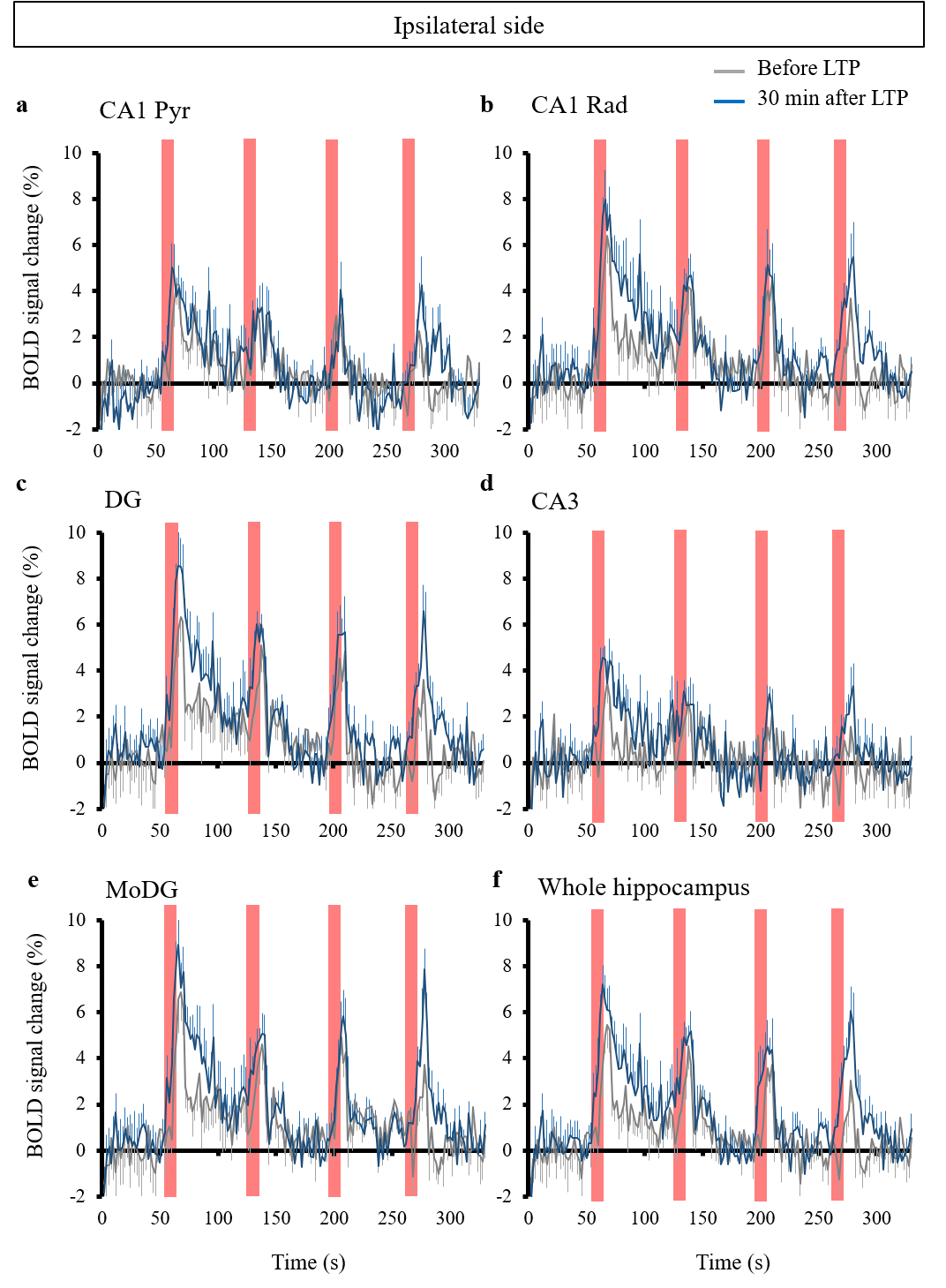


**Supplementary Fig. S2 BOLD time courses of the ipsilateral hippocampus.**

The entire BOLD time courses in the CA1 Pyr (a), CA1 Rad (b), DG (c), CA3 (d), MoDG (e), and whole hippocampus (f) before and after LTP induction. The red boxes correspond to periods of electrical stimulation at the perforant pathway. The bar plots exhibit mean ±sem.


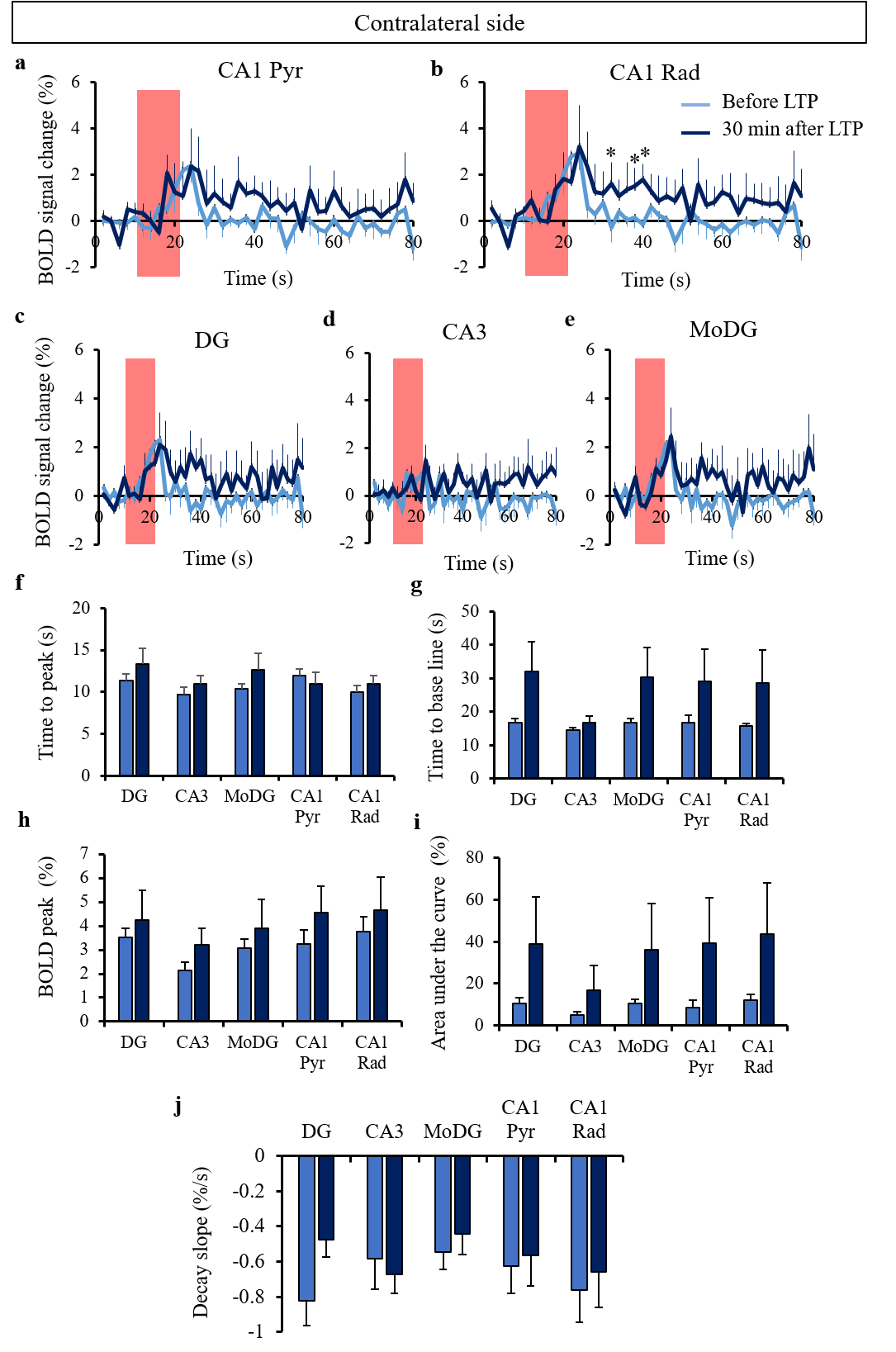


**Supplementary Fig. S3 BOLD time courses and response parameters in the contralateral hippocampus.**

BOLD time courses at CA1 Pyr (a), CA1 Rad (b), DG (c), CA3 (d), and MoDG (e) in the contralateral hippocampus before and after LTP induction. Time to peak (f), time to base line (g), BOLD peak (h), area under the curve (i), and decay slope (j) at DG, CA3, MoDG, CA1 Pyr, and CA1 Rad before and after LTP induction. The red boxes represent electrical stimulation periods at the perforant pathway. The bar plots exhibit mean±sem. * p<0.05 (paired t-test). The interaction between time and LTP effects of the two-way repeated ANOVA showed no significance.


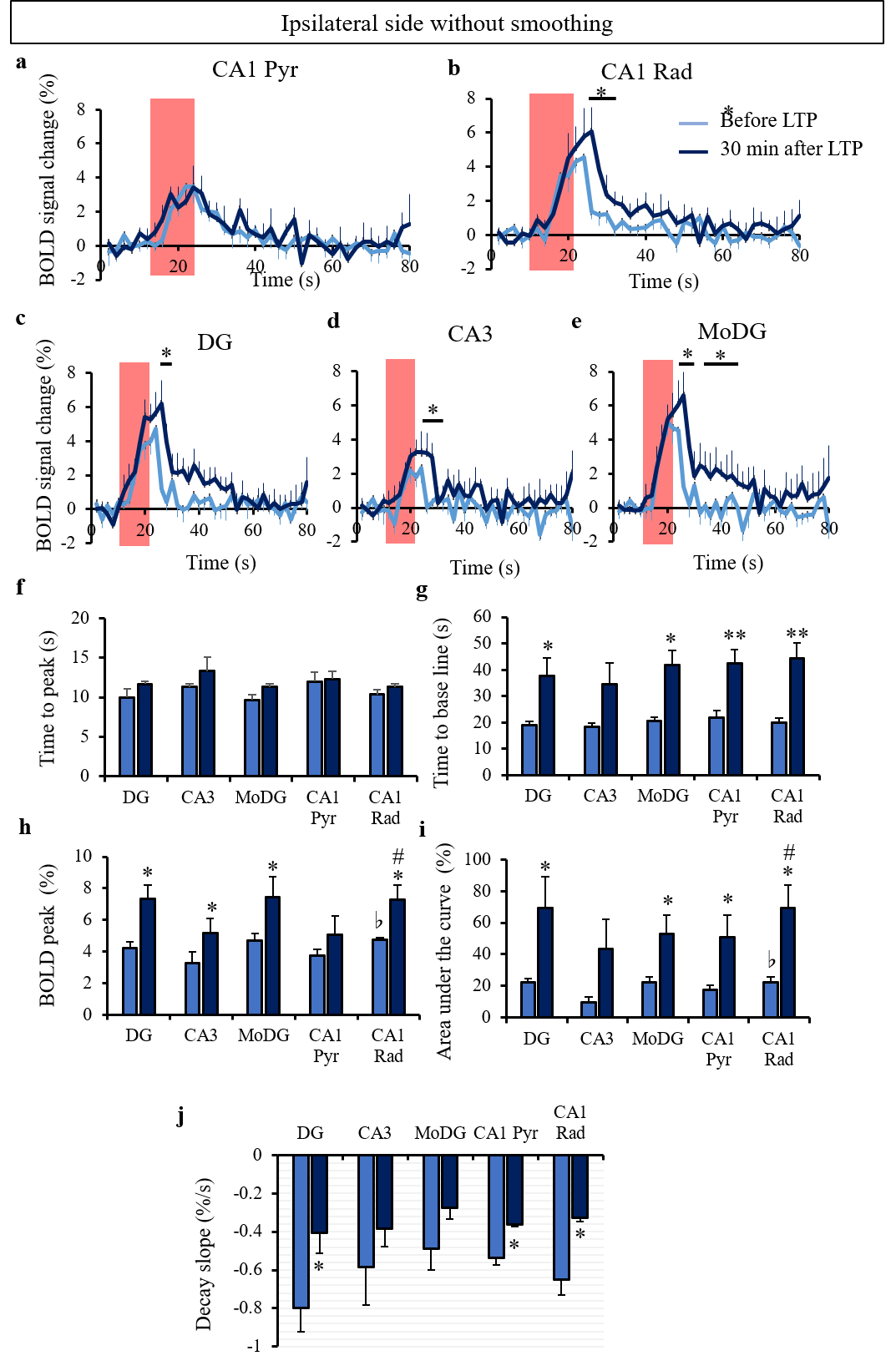


**Supplementary Fig. S4** **BOLD time courses and response parameters in the ipsilateral hippocampus without performing smoothing.**

(a-e) BOLD time courses of CA1 Pyr (a), CA1 Rad (b), DG (c), CA3 (d), and MoDG (e) before and after LTP induction. The red boxes correspond to periods of electrical stimulation at the perforant pathway. The bar plots exhibit mean ±sem. * p<0.05 (paired t-test). The interaction between BOLD time course and LTP effects of two-way repeated ANOVA showed no significance. (f-j) Time to peak (f), time to baseline (g), BOLD peak (h), area under the curve (i), and decay slope (j) at DG, CA3, MoDG, CA1 Pyr, and CA1 Rad before and after LTP induction. *: p<0.05, **: p<0.01 (paired t-test after versus before LTP at each region), ♭: p<0.05 (paired t-test Rad versus Pyr before LTP), and #: p<0.05 (paired t-test Rad versus Pyr 30 min after LTP).
